# Increased expression of CELSR3 indicates a poor prognostic factor for Prostate Cancer

**DOI:** 10.7150/jca.49567

**Published:** 2021-01-01

**Authors:** Xuanrong Chen, Qianwang Ma, Yixi Liu, Hanling Li, Zihao Liu, Zheng Zhang, Yuanjie Niu, Zhiqun Shang

**Affiliations:** Department of Urology, Tianjin Institute of Urology, The second hospital of Tianjin Medical University, Tianjin, 300211, China.

**Keywords:** Prostate cancer, flamingo subfamily, prognostic factor, Bioinformatics

## Abstract

**Background:** Cadherin EGF LAG Seven-Pass G-Type Receptor 3 (CELSR3) gene was reported to be overexpressed in various human cancers and involved in the regulation of neurite-dependent neurite outgrowth and may play a role in tumor formation. However, the clinical significance of CELSR3 in prostate cancer (PCa) has not been fully studied.

**Methods:** The expression of CELSR3 was detected by crossover analysis of the public datasets and cell lines. MTT assay and migration assay were performed to evaluate the cells' physiological functioning. Co-expressed genes and enrichment analysis was performed to investigate the biological significance of CELSR3 in PCa. Quantitative real-time polymerase chain reaction was used to detect the expression levels of hub genes (CENPE, CENPA, CDC20, NUF2, ESPL1, PLK1) related to CELSR3.

**Results:** We found a significant increase in CELSR3 expression in PCa patients and cell lines. Furthermore, immunohistochemical analysis showed that CELSR3 protein expression was significantly more highly expressed in the PCa tissues compared to the non-cancerous PCa tissues. CELSR3 downregulation significantly suppressed cell proliferation and migration potential. CELSR3-related hub genes (CENPE, CENPA, CDC20, NUF2, ESPL1, PLK1) were selected and the functions of these hub genes showed that the function of CELSR3 was closely related to the cell cycle-related signaling pathways. The upregulation of CELSR3 mRNA expression in the PCa tissues significantly correlated with the presence of high serum PSA levels, high pathological stage, high Gleason score, short overall survival time and short disease-free survival time.

**Conclusion:** Our data suggest that CELSR3 may play an important role in the progression of PCa. More importantly, an increase in CELSR3 expression may be indicative of poor disease-free survival and poor prognosis in PCa patients.

## Introduction

Prostate cancer (PCa) has become the second most common cancer in men, and both morbidity and mortality are on the rise in recent years^1^. As a heterogeneous disease, the occurrence of PCa may be affected by multiple factors such as genes, cellular context, and environment^2^. Gene expression profiling using high-throughput platforms is increasingly used as an effective tool for analysing tumor progression mechanisms. There are a variety of gene expression databases and profiles for prostate cancer established using microarray and sequencing technology^3^. Cumulative evidence suggests that multiple genes and signalling pathways are involved in the carcinogenesis, progression, and recurrence of PCa. However, its tumor-related mechanisms have not been fully clarified. Therefore, it is crucial to identify efficient biomarkers to better predict the diagnosis and prognosis of PCa.

Cadherin EGF LAG seven-pass G-type receptors 1, 2 and 3 (CELSR1-3) constitute three atypical cadherin families with multiple functions in epithelial cells and the nervous system^4^, ^5^. As we know, cadherin is a calcium-dependent transmembrane glycoprotein that is typically characterized by an extracellular calcium binding domain that plays an important role in embryonic development and synapse formation^6^. CELSR3 is an important signaling molecule in the INT-1/planar cell polarity (WNT/PCP) pathway, and this pathway is involved in controlling tissue polarity and cell migration^7^. According to reports, the CELSR3 gene is overexpressed in various human cancers, including brain tumors, ovarian cancer, pancreatic cancer, liver cancer, colorectal cancer and cervical cancer^8^-^11^. CELSR3 was specifically expressed in adult brain tumors. Erkan et al. showed that CELSR3 was selectively up-regulated in the stellate cells of liver and pancreas cancer^11^. Asad et al. found that CELSR3 was expressed more strongly in Stem-A than non-Stem-A ovarian cancer and that CELSR3 might promote Stem-A ovarian cancer invasiveness by regulating cell proliferation and cell cycle progression through a non-canonical Wnt/PCP pathway^8^. Through ceRNA network analysis, Pan et al. demonstrated that CELSR3 was a prognostic gene for the overall survival of head and neck squamous cell carcinoma^12^. Gu et al. showed that CELSR3 mRNA was an independent prognostic factor for the overall survival of hepatocellular carcinoma patients^13^. Gene set enrichment analysis revealed that a high CELSR3 mRNA expression phenotype related to somatic diversification of immune receptors, endonuclease activity, DNA repair complex, and somatic cell DNA recombination^13^, ^14^. In all, CELSR3 was found to be an oncogene that promoted malignant progression of the tumor. However, the clinical value and role of CELSR3 in PCa has not been fully elucidated.

In this study, we found that high expression of CELSR3 may be a potentially effective biomarker for PCa. Cox proportional hazards regression model analysis showed that increased expression of CELSR3 is closely related to the clinical pathology of PCa. This expression pattern of CELSR3 can be used as a prognostic biomarker and is associated with poor prognosis in patients with prostate cancer.

## Materials and Methods

### Public datasets and immunohistochemistry analysis

The clinical information and expression profiles were obtained from the MSKCC, PRAD, and PCTA datasets and used to conduct the analysis^3^, ^15^, ^16^. The main parameters of the public datasets were summarized in Table 1. For prostate cancer specimen, CELSR3 antibody (Atlas Antibodies, Cat#HPA062866, RRID: AB_2684886) was used. Immunohistochemical staining of human PCa cells shows moderate cytoplasmic or membranous positivity in the luminal cancer cells in the Human Protein Atlas database 17, 18.

### Cell culture and transfection

The BPH1, LNCaP, C42, 22RV1 cell lines were obtained from the American Type Culture Collection (ATCC) and grown in RPMI 1640 (Gibco, ThermoFisher, USA) supplemented with 10% Fetal bovine serum (FBS) (Gibco, ThermoFisher, USA) in 5% CO^2^ at 37°C. CELSR3 silencing was performed using two custom made siRNAs targeting CELSR3 mRNA region (siCELSR3 #1 sense: GCAAUAGCCGUGGACACUUTT, siCELSR3 #1 antisense: AAGUGUCCACGGCUAUUGCTT and siCELSR3 #2 sense: CCAGUACACUUUCCAGAAUTT, siCELSR3 #2 antisense: AUUCUGGAAAGUGUACUGGTT) and one scramble control siRNA (Genepharma, China) and transfected by Lipofectamine™ 3000 Transfection Reagent followed the manufacturer's instructions.

### RNA extraction and Quantitative RT-PCR analysis

Total RNA was isolated from cells using TRIzol reagent (Invitrogen, USA) according to the manufacturer's instructions. Quantitative RT-PCR (qRT-PCR) was performed on an ABI 7900 96 HT Series PCR Instrument (Applied Biosystem, ThermoFisher, USA) using FastStart Universal SYBR Green Master (ROX) (Roche, Sigma-Aldrich). Gene expression levels were determined by the Ct method and further normalized to GAPDH levels. The sequences of the primers used were listed in Table S1.

### Cell growth assay

Cell growth was monitored by the 3-(4,5-dimethylthiazol-2-yl)-2,5-diphe-nyltetrazolium bromide proliferation assay. 22RV1 cells were seeded into a 96-well plate at a density of 2000 cells per well. After for 24 hours of incubation, cell proliferation ability was evaluated every 24 hours for three consecutive days. Each experiment was performed in triplicate and repeated at least three times.

### Transwell migration assay

For migration detection, 100 ul of 1640 medium containing 2 × 10^4^ cells were added to the upper chamber of a 24-well plate, and 500 μl of 1640 medium containing 20% FBS was supplemented in the lower chamber. After incubating for 48 hours in a 37 ° C incubator, cells were washed twice with PBS solution, fixed with methanol for 20 minutes at room temperature, stained with 0.1% crystal violet solution, and then observed under an optical microscope. Each experiment was performed in triplicate and repeated at least three times.

### Co-expressed genes and enrichment analysis

Co-expressed genes of CELSR3 may play similar biological functions in cooperation with CELSR3 in PCa. From the cBioPortal for Cancer Genomics portal (http://cbioportal.org), we conducted a Spearman correlation analysis and screened the co-expressed genes of CELSR3 in the PRAD dataset. CELSR3 and its most significant positively co-expressed genes (r≥0.4, p<0.01, q<0.01) were used for further enrichment analysis in Metascape (https://metascape.org). All genes in the genome were used as background information for the analysis. The Molecular Complex Detection (MCODE) algorithm was used to conduct protein-protein interaction enrichment analysis.

### Statistical analysis

Statistical analysis was performed using SPSS software (version 17.0; SPSS Inc., Chicago, Illinois, USA). The experimental results were evaluated using an independent Student t-test and the data were presented as mean ± SD (standard deviation). Briefly, we performed a contingency table to investigate the relationship between CELSR3 expression and clinicopathological variables in PCa patients. Survival analysis was performed using the Kaplan-Meier curve method 19. A Cox proportional hazard regression model was performed to determine the prognostic value of CELSR3 expression for the biochemical recurrence-free survival and overall survival. First, we analyzed the association between biochemical-free recurrence and potential prognostic factors, including the Gleason score, PSA, pathology, age, and clinical stage, taking into account one factor at a time. Second, multivariate Cox analysis was applied to the backward (stepwise) process, which always forces CELSR3 expression into the model. A P value <0.05 was considered statistically significant.

## Results

### Increased expression of CELSR3 in human PCa tissues

**Figure 1:**
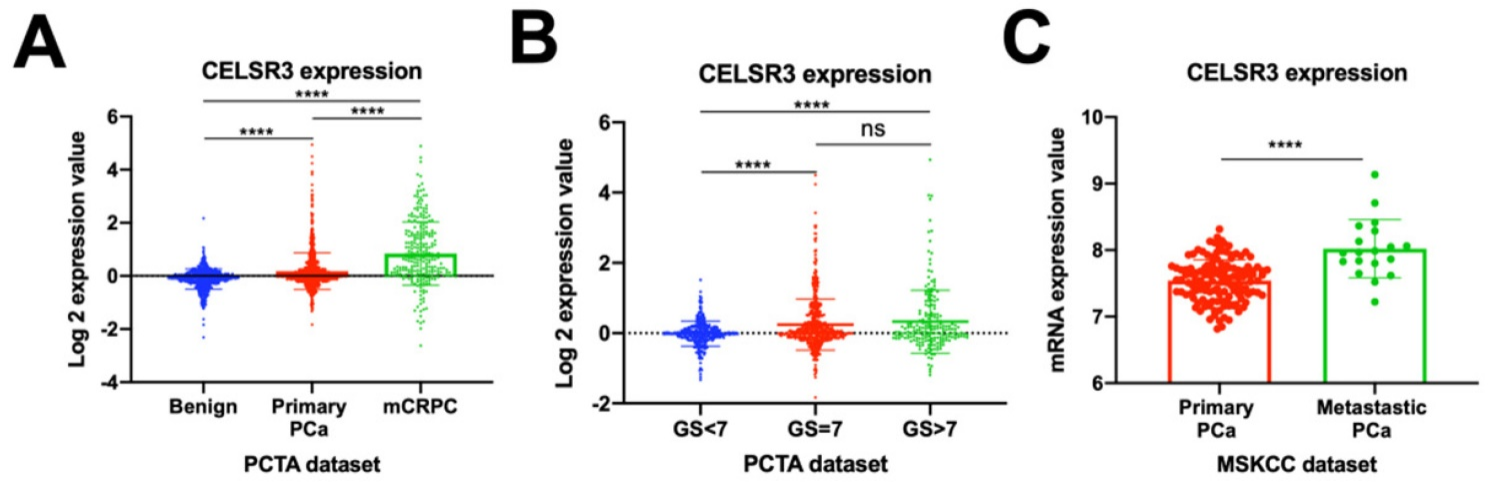
CELSR3 mRNA expression in PCa patients and PCa cell lines. (A) CELSR3 mRNA expression in the PCTA dataset. (B) CELSR3 mRNA expression in primary PCa patients. (C) CELSR3 gene expression profiles in the MSKCC dataset. ***P<0.001; **** P<0.0001; ns, no significance. PCa, prostate cancer; mCRPC, metastatic castration-resistant prostate cancer; GS, Gleason score.

Previous studies have reported that the atypical cadherin family was critical in the development and progression of tumors 7. First, we investigated the CELSR3 gene expression profiles in the PCTA dataset (Figure 1A and 1B). We confirmed that CELSR3 was significantly upregulated in PCa (primary PCa and metastatic castration-resistant prostate cancer) when compared to benign prostatic hyperplasia in the PCTA dataset (Figure 1A; P<0.001). Furthermore, the expression of CELSR3 was significantly increased in mCRPC compared to primary PCa, and this expression pattern was associated with tumor progression (P < 0.001). To further investigate the expression pattern of CELSR3 in primary PCa, we grouped patients according to the Gleason score (GS) and compared the relationship between CELSR3 expression and Gleason score (Figure 1B). In Figure 1B, patients with Gleason score (GS) >7 had higher CELSR3 expression levels than those with GS<7 (P <0.001). In addition, those with GS=7 had higher expression levels of CELSR3 than patients with GS<7 (P <0.001). We then analyzed the expression profile of the CELSR3 gene in the MSKCC dataset to further assess this dysregulated change (Figure 1C). We confirmed that in the MSKCC dataset, metastatic PCa significantly up-regulated CELSR3 compared to primary PCa (P < 0.001).

The Human Protein Atlas is a protein expression database of all the human proteins in cells, tissues and organs 18. We investigated the CELSR3 protein expression in this database (Figure 3). Clearly, PCa patient (patient ID: 5416) showed higher levels of CELSR3 protein expression than benign prostate sites (Figure 3A). High-grade PCa patient (patient ID: 2823) showed high-intensity level of CELSR3 protein expression (Figure 3B). This suggests that the expression pattern of CELSR3 protein may be similar to the high mRNA expression pattern found in PCa patients.

### Increased expression of CELSR3 in human PCa cell lines

To cross-validate the findings in the PCa patient dataset, we performed quantitative RT-PCR analysis of CELSR3 mRNA expression in three PCa cell lines (LNCaP, C42, 22RV1) and one benign cell line (BPH1). We confirmed that CELSR3 mRNA was significantly upregulated in PCa cancer cell lines when compared to BPH1 (Figure 1D, P<0.05). In particular, 22RV1 is a human prostate cancer epithelial cell line derived from xenografts (continuous passage in mice that have undergone prostate cancer regression and relapse after grafting of their father's androgen-reliable CWR22) 20, 21. The mRNA expression level of CELSR3 was highest in the 22RV1 cell line compared to other cancer cell lines, which was also consistent with the highly malignant characteristics of this cell line.

### Correlation between CELSR3 and cell physiological function in prostate cancer cells

**Figure 2:**
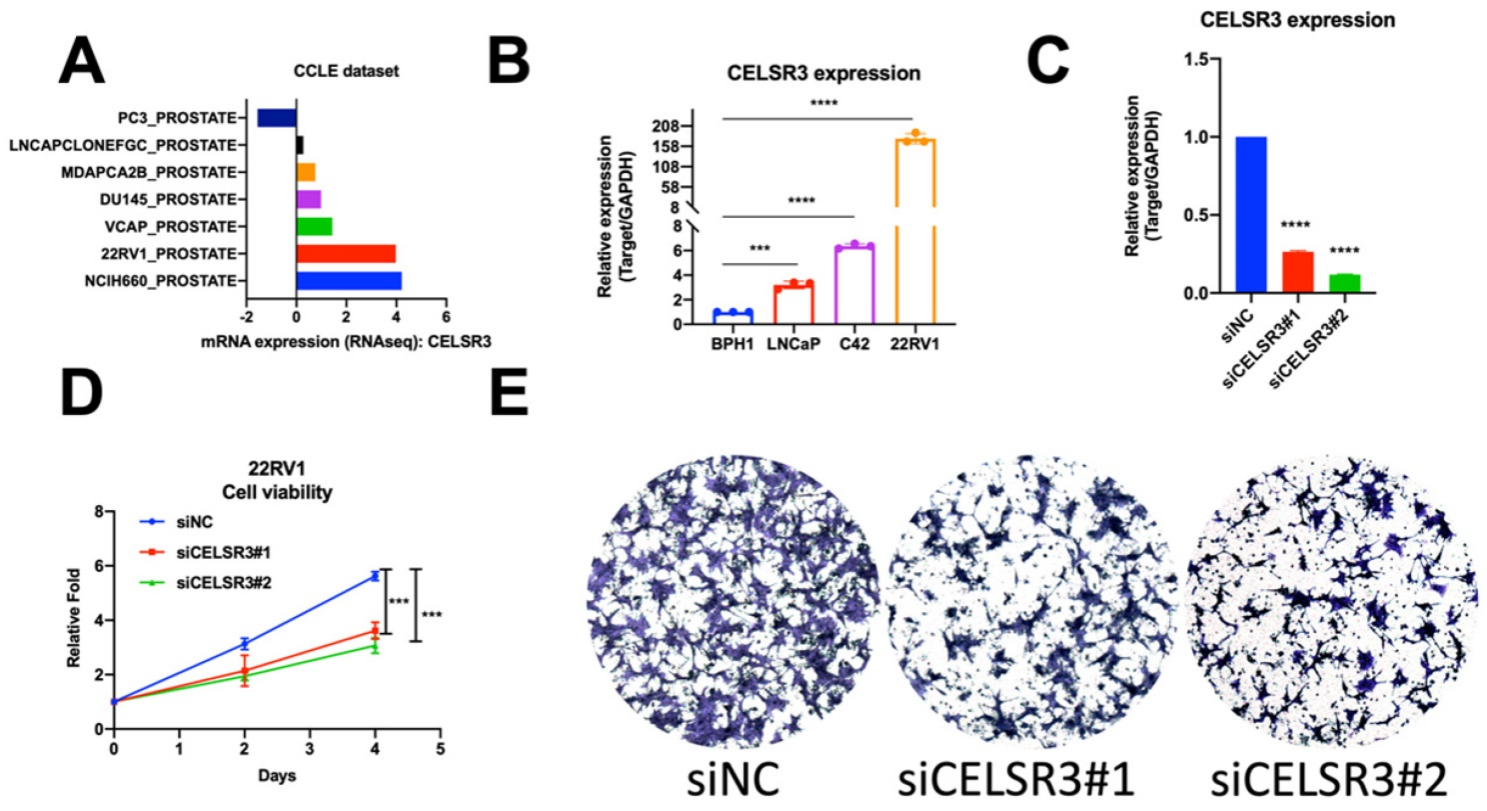
Cell physiological function of CELSR3 in PCa. (A) CELSR3 mRNA expression in the CCLE dataset. (B) CELSR3 mRNA expression in PCa cell lines. (C) CELSR3 knockout efficacy. The expression of CELSR3 in siRNA target cells was remarkably decreased. (D) The proliferation ability in siRNA target cells was significantly suppressed compared to control cells. (E) The cell migration capacity in siRNA target cells was significantly suppressed compared to control cells. ***P<0.001; **** P<0.0001

To investigate the function of CELSR3 in PCa cells, we used a siRNA-mediated assay to knockdown the expression of CELSR3. As shown in Figure 2C, the expression of CELSR3 was prominently downregulated in the 22RV1 cell line, which confirmed the knockdown efficacy by qRT-PCR. In Figure 2D, the cell proliferation viability was detected by the MTT assay. In the CELSR3 knockdown group, cells grew faster than the negative control group, which suggested that CELSR3 may be able to promote cell proliferation in PCa. In addition, using a Transwell assay, we confirmed that CELSR3 may be able to promote cell migration in PCa, because the migration ability of cells in the CELSR3 knockdown group showed significant inhibition compared to negative control cells (Figure 2E).

### Enrichment analysis and protein network

In order to further study the possible biological function of the CELSR3 gene in PCa, we performed gene co-expression analysis through cBioPortal (the PRAD dataset), because co-expressed genes usually have similar functions and have certain biological significance. We chose the most significantly positively co-expressed genes (r≥0.4, p<0.01, q<0.01) to identify the differentially activated signaling pathways or biological functions (Table 4). Metascape provides a biologist-oriented resource for gene enrichment analysis. As shown in Figure 4A and Table 5, Metascape-based pathway and process enrichment analysis revealed 15 clusters, which were chosen to be represented by the most statistically significant terms in the cluster (p-value < 0.01, minimum gene counts: 3, and enrichment factor > 1.5). The mitotic sister chromatid segregation, mitotic cell cycle phase transition, cell cycle, mitotic cytokinesis, and PID FOXM1 pathway were the top 15 clusters enriched in phenotypes with positively co-expressed genes of CELSR3. Protein-protein interaction enrichment analysis was performed with the Molecular Complex Detection (MCODE) algorithm to identify densely connected network components. The MCODE component (MCODE_1) was gathered and shown in Figure 4B and 4C. The MCODE_1 component contains 6 hub genes (CENPE, CENPA, CDC20, NUF2, ESPL1, PLK1). We also demonstrated that CELSR3 was positively correlated to the expression of CDC20 (Spearman r=0.44, p<0.05), CENPA (Spearman r=0.41, p<0.05), CENPE (Spearman r=0.43, p<0.05), ESPL1 (Spearman r=0.40, p<0.05), PLK1 (Spearman r=0.43, p<0.05), and NUF2 (Spearman r=0.41, p<0.05), respectively (Figure 5 A-F). In addition, by siRNA-mediated knockdown assay, we confirmed that these hub genes were down-regulated in the CELSR3 knockdown group (Figure 5 G). The functions of these hub genes, once again, showed that the function of CELSR3 was closely related to the cell cycle-related signaling pathway, which was also consistent with the in vitro phenotype experiments described in the present study.

### Relationship between CELSR3 mRNA expression and clinical features of PCa

**Figure 3:**
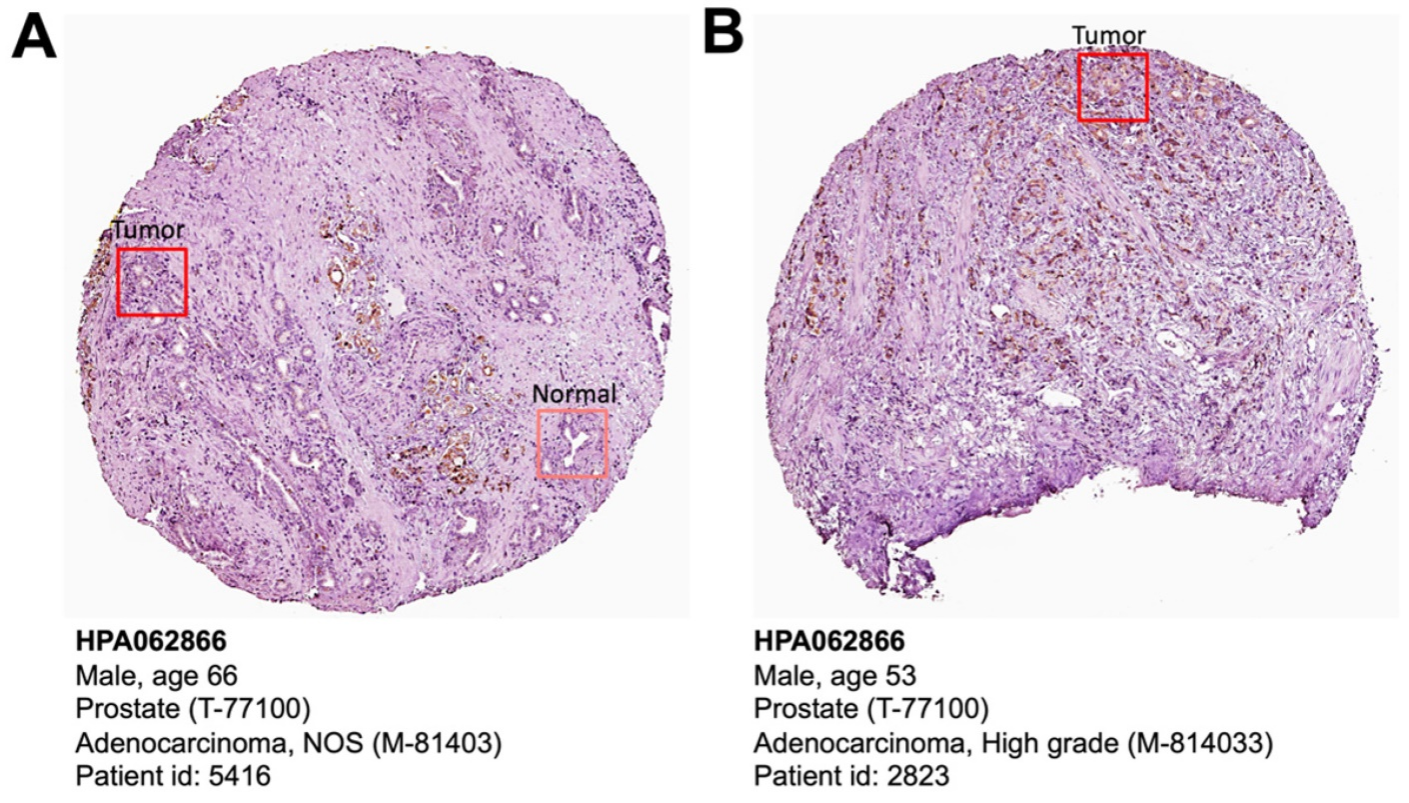
CELSR3 protein expression in PCa patient specimens. (A) Immunohistochemical staining showed expression of CELSR3 in normal prostate and PCa tissues. (B) Immunohistochemical staining showed expression of CELSR3 in a high-grade PCa patient. The pictures were taken from the HPA website (https://www.proteinatlas.org/ENSG00000008300-CELSR3/pathology/prostate+cancer#ihc).

**Figure 4:**
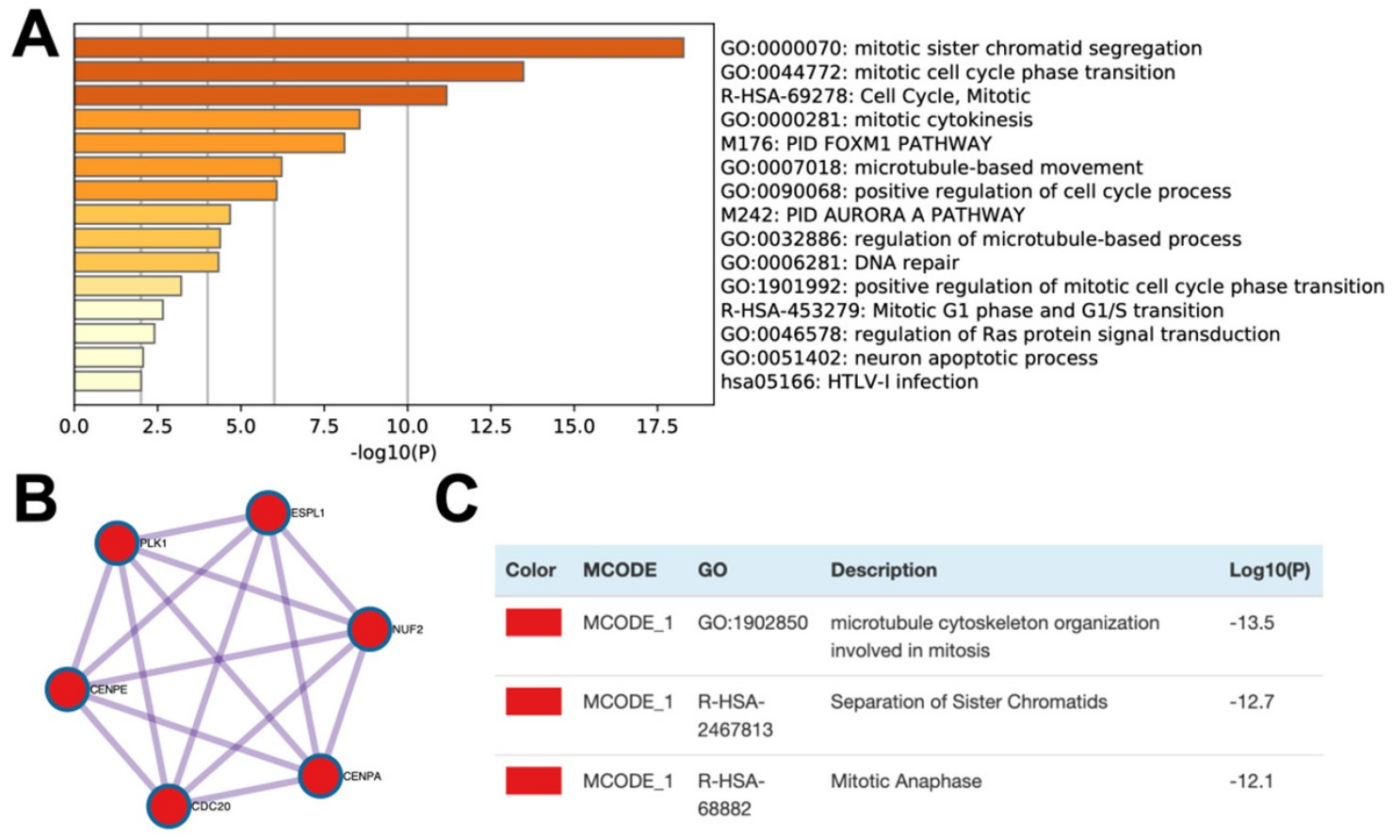
Enrichment analysis and protein networks of the co-expressed genes. (A) Bar graph of enriched terms across the co-expressed genes. (B and C) MCODE components of the co-expressed genes.

**Figure 5:**
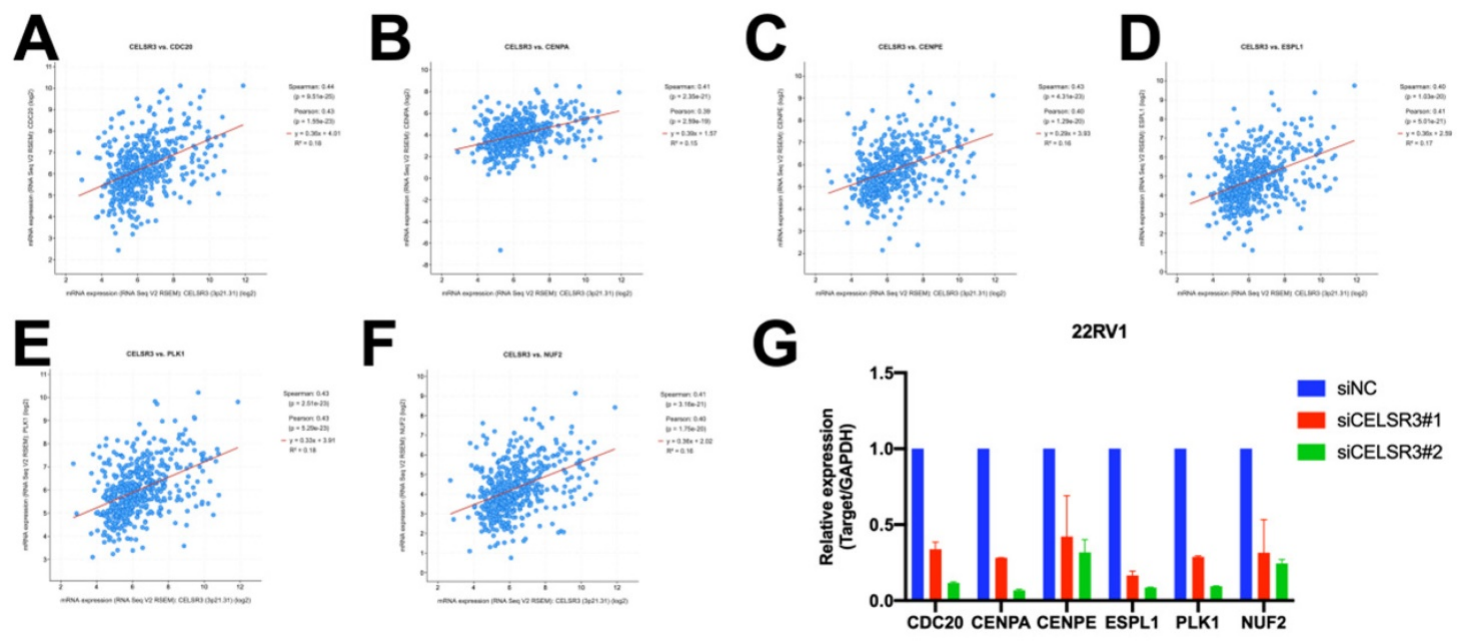
The correlation between CELSR3 and the hub genes. (A-D) The expression of CENPE, CENPA, CDC20, NUF2, ESPL1, PLK1 was significantly correlated to CELSR3, respectively. (G) The expression of CENPE, CENPA, CDC20, NUF2, ESPL1, and PLK1 were down-regulated in the CELSR3 knockdown group.

As with overall survival (OS), disease-free survival is also critical for PCa patients. Disease-free survival (DFS), or biochemical recurrence, was defined as the surrogate endpoint after radical prostatectomy. The clinical use of DFS is to identify those who would gain benefit from early initiation of salvage treatment with additional therapy as early as possible 22. We performed a Kaplan-Meier curve method to assess the relationship between CELSR3 expression levels in the PRAD dataset and OS and DFS (Figure 3A and 3B). According to the GEPIA website, an interactive web server for analyzing the RNA sequencing expression data of the Cancer Genome Atlas (TCGA) and the Genotype-Tissue Expression (GTEx) projects, we used the quartile CELSR3 mRNA expression level as the cutoff point in order to split all samples into CELSR3 high (n = 123, in the PRAD dataset) and CELSR3 low (n = 122, in the PRAD dataset) groups. We performed statistically significant validation of survival analyses in both the high CELSR3 group and the low CELSR3 group. Patients in the high CELSR3 group showed a shorter probability of overall survival compared to the low CELSR3 group (Figure 3A; p=0.011). And the DFS was also statistically significant (Figure 3B; p=0.0024). This meant that patients with high expression of CELSR3 had shorter disease-free survival time and overall survival probability.

Moreover, we investigated the connection of CELSR3 mRNA expression with multiple clinic‑pathological characteristics according to the PRAD dataset (Table 2). The results showed that patients with high serum PSA levels (P<0.002), high pathological stage (≥T3A; P<0.001), high Gleason score (GS≥8; P<0.001), short overall survival time (P<0.001) and short disease-free survival time (P<0.05) had higher levels of CELSR3 expression than patients with lower serum PSA levels, lower pathological stage, lower Gleason score (GS<8), longer over survival time and longer disease-free survival time.

### High expression of CELSR3 as a prognostic factor of human PCa

To further investigate the prognostic value of CELSR3 in PCa, we performed a univariate and multivariate Cox proportional hazard regression to validate the clinical prognostic value of CELSR3 in the PRAD dataset (Table 3). The results showed that CELSR3 (P=0.006), the GS (P<0.001), PSA (P=0.002) and pathological stage (P<0.001) were appropriate for being considered as prognosis factors for DFS via univariate analysis. We confirmed that CELSR3 (P<0.001), the GS (P=0.007) and PSA (P=0.036) were appropriate for being considered as prognosis factors for OS via univariate analysis. Next, we performed multi‑variate analysis and found that GS was potentially independent factors for predicting shorter both DFS (P<0.001) and OS (P=0.027) survival. Because of the number of deceased in the PRAD dataset (only 10 patients died in total), the clinical prognostic value of CELSR3 in OS still needs to be further studied in the future.

## Discussion

The treatment of PCa imposes a huge economic burden on patients and society^23^. Although many PCa patients can benefit from techniques such as prostate-specific antigen (PSA) screening, the time between tumor progression to biochemical recurrence and tumor metastasis remains unpredictable^24^. Most PCa patients eventually relapse due to castration-resistant prostate cancer, with tumor metastasis and more malignant invasive events^25^. These patients will inevitably have a poor prognosis and death because there is no effective treatment^26^. Therefore, it is crucial to find reliable biomarkers to predict the occurrence of adverse prognostic events such as recurrence and metastasis^27^. To the best of our knowledge, increased expression of CELSR3 has been reported in brain tumors, ovarian cancer, pancreatic cancer, liver cancer and cervical cancer^8^, ^10^. However, there are no relevant studies on CELSR3 in the field of prostate tumor research.

The CELSR3 gene belongs to the flamingo subfamily and is included in the cadherin superfamily^6^. Flamingo cadherin is composed of a non-classical cadherin that does not interact with catenin. Flamingo cadherin is a plasma membrane protein containing seven epidermal growth factor-like repeats, nine cadherin domains and two laminin A G repeats in its extracellular domain. It also has seven transmembrane domains, which are typical of this subfamily. It has been reported that CELSR3 may have an important role in cell/cell signaling during the nervous system formation and in tumor formation^6^, ^9^, ^13^. The research on CELSR3 focuses on the influence of CELSR3 on the function of the nervous system^28^. They found that the CELSR3 protein plays an important role in the migration of cortical neurons and the formation of neuron axons and dendrites.

Erkan et al. suggested that tissue fibrosis was an important component of chronic inflammation of the liver and pancreas and pancreatic cancer, while multiple activated cells played a key role in fiber formation^11^. The results showed that CELSR3 was highly expressed in stellate cells of pancreatic cancer and could be a selective target providing a favorable therapeutic strategy. To determine the molecular biological basis of colorectal cancer metastasis, Goryca et al. performed a whole-exome and transcriptome sequencing of seven liver metastases and matched primary and normal tissues^9^. The authors showed that CELSR3 had a unique gene variant in metastatic patients, suggesting that CELSR3 was involved in the metastasis of colorectal cancer by altering the structure of the gene or its mutation.

In the present study, we found that CELSR3 was highly expressed in PCa in the PCTA dataset, and through further survival analysis, we believed that CELSR3 may be a very important oncogene. We investigated the gene expression profile of CELSR3 in the MSKCC dataset and confirmed a significant increase in the expression of CELSR3 in PCa and PCa cell lines, especially those with higher Gleason scores (GS > 7). To study the clinical value and potential molecular mechanisms of CELSR3 in PCa, we investigated the role of CELSR3 in the proliferation and migration of 22RV1 cells (a PCa cell line) by in vitro experiments. Using enrichment analysis and protein network analysis, we investigated the possible molecular mechanism of CELSR3 in PCa. Six hub genes (CENPE, CENPA, CDC20, NUF2, ESPL1, PLK1) were selected and the functions of these hub genes showed that the function of CELSR3 was closely related to the cell cycle-related signaling pathway, which was also consistent with the in vitro phenotype experiments. To further validate the representativeness of this prognostic value in PCa, we performed the Kaplan-Meier survival analysis and Cox proportional hazards regression model. Our results suggested that increased expression of CELSR3 was significantly associated with poor DFS and OS in patients. Gu et al. showed that in hepatocellular carcinoma (HCC), mRNA expression of CELSR3 was also increased in tissue samples and cell lines^13^. Through bioinformatics analysis, the pathways in which CELSR3 was highly expressed were enriched in the BRCA pathway, E2F targets, hallmark G2/M checkpoint and hallmark spermatogenesis. Notably, these data suggested that these pathways might be key pathways for CELSR3 regulation of liver cancer. We utilized in vitro experiments to validate the biological functions of CELSR3 in the cell cycle-related signaling pathways. In summary, CELSR3 is thought to be a malignant oncogene. However, in this study, in vivo experiments have not been conducted to validate the role of CELSR3 in PCa, and in vivo experiment is considered to be a powerful method to investigate this potential role of CELSR3 in PCa.

Together, our data demonstrate the important role of CELSR3 in the prognosis of PCa for the first time. An increase in CELSR3 expression may be used as a prognostic biomarker for PCa and predict a poor prognosis (both DFS and OS) in patients. CELSR3 may serve as a therapeutic target for PCa, however the possible molecular mechanisms and expression patterns of CELSR3 in PCa still need to be further investigated.

## Supplementary Material

Supplementary table.

## Figures and Tables

**Figure 6:**
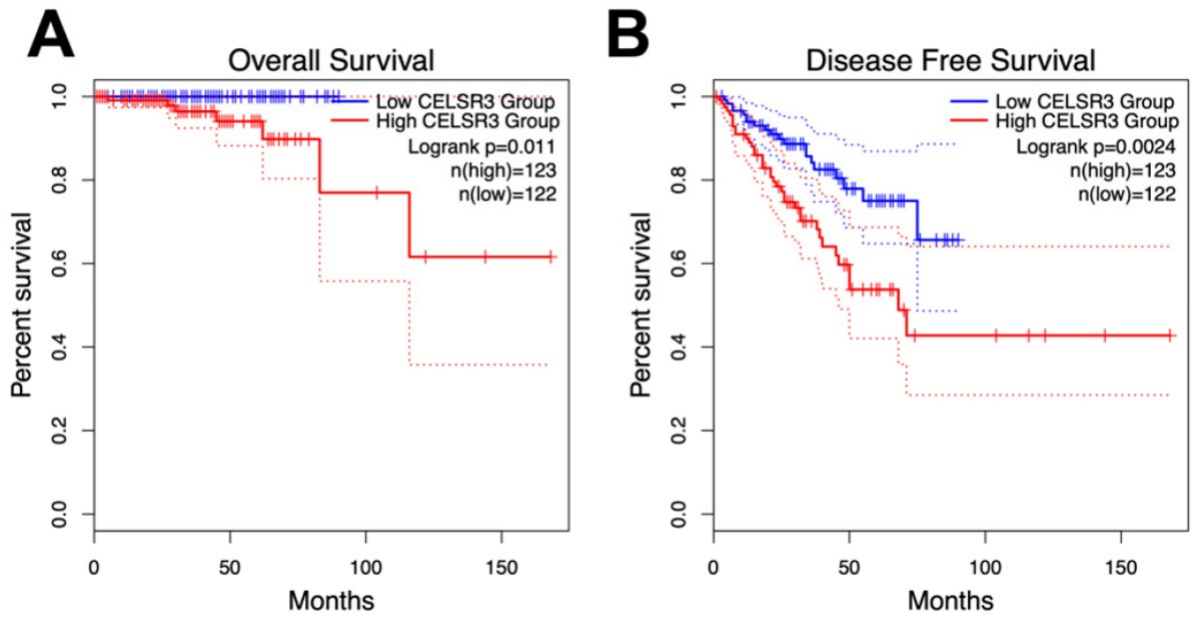
Survival analysis of CELSR3 expression and gene alterations in terms of the disease-free survival (DFS) and overall survival (OS). The CELSR3 mRNA expression level showed a prognostic value in OS (A) and in DFS (B) in the PRAD dataset.

**Table 1:** The main parameters of the public datasets.

Dataset	Characteristic	Reference
the MSKCC dataset	218 prostate tumors with 181 primaries tumors and 37 metastases tumors	3
the PRAD dataset	497 primary prostate cancer samples with mRNA and clinicopathological characteristics^*^	16
the PCTA dataset	1,321 clinical specimens from 38 PCa cohorts	15

*, the PRAD dataset refers to the Prostate Adenocarcinoma (TCGA, TCGA Provisional) dataset.

**Table 2:** Connection of CELSR3 expression with clinicopathological characteristics of PCa in the PRAD dataset.

		The PRAD dataset	
*CELSR3 expression*	n	Mean ± SD	*P*
*CELSR3 expression*			
Benign	-		
PCa	497	-0.006±0.992	-
*Serum PSA*			
< 4 (ng/ml)	413	-0.057±0.796	
≥ 4 (ng/ml)	27	0.550±2.415	**<0.002**
*Age*			
< 66 years	354	0.003±1.067	
≥ 66 years	143	-0.027±0.781	0.999
*Pathological stage*			
< T3A	187	-0.227±0.477	
≥ T3A	303	0.099±0.984	**<0.001**
*Gleason score*			
< 8	292	-0.220±0.454	
≥8	205	0.299±1.394	**<0.001**
*Overall survival*			
Alive	487	-0.035±0.836	
Decease	10	1.391±3.783	**<0.001**
*Disease-free survival*			
Disease Free	400	-0.075±0.786	
Recurred/Progressed	91	0.173±1.033	**<0.05**

- Lack of relative information or no results

**Table 3:** Prognostic value of CELSR3 mRNA expression level for the disease-free survival (DFS) and overall survival (OS) via Cox proportional hazards model.

	DFS		OS	
	Hazard ratio (95% CI)	*P*	Hazard ratio (95% CI)	*P*
**Univariate analysis**				
CELSR3 mRNA	1.292(1.077-1.550)	**0.006**	1.785(1.341-2.376)	**<0.001**
Gleason score	2.190(1.763-2.721)	**<0.001**	2.973(1.342-6.585)	**0.007**
PSA	1.048(1.017-1.080)	**0.002**	1.062(1.004-1.124)	**0.036**
Pathological stage	2.615(1.733-3.945)	**<0.001**	2.245(0.600-8.403)	0.230
Age	1.027(0.995-1.060)	0.095	1.053(0.956-1.161)	0.294
**Multivariate analysis**				
CELSR3 mRNA	1.143(0.571-2.288)	0.614	4.591(1.584-13.309)	0.835
Gleason score	1.944(1.533-2.465)	**<0.001**	2.361(1.104-5.049)	**0.027**
PSA	1.043(0.980-1.110)	0.155	1.005(1.001-1.009)	0.212
Pathological stage	1.702(1.043-2.778)	**0.033**	2.198(1.281-3.770)	0.987
Age	1.053(0.938-1.181)	0.705	1.024(0.974-1.077)	0.438

**Table 4:** Genes positively correlated with CELSR3 in mRNA expression in the PRAD dataset. (Cutoff: Spearman's correlation r≥0.4, p<0.01, q<0.01)

Correlated Gene	Spearman's correlation r value	p-Value	q-Value
SPAG5	0.48337077	4.10E-30	8.26E-26
MMP11	0.45691182	1.07E-26	1.08E-22
KRTAP5-1	0.44564415	2.49E-25	1.25E-21
MYBL2	0.44276489	5.46E-25	2.03E-21
B4GALNT4	0.44157118	7.54E-25	2.03E-21
TCF3	0.44148613	7.72E-25	2.03E-21
CDC20	0.4407113	9.51E-25	2.13E-21
NBEAL2	0.44016103	1.10E-24	2.13E-21
MGAT4B	0.4391724	1.44E-24	2.42E-21
TROAP	0.43792972	2.01E-24	3.11E-21
GTSE1	0.43149301	1.10E-23	1.48E-20
PLK1	0.42832255	2.51E-23	3.16E-20
CENPE	0.42622476	4.31E-23	4.94E-20
FAM72B	0.42613757	4.41E-23	4.94E-20
KIF14	0.42545536	5.26E-23	5.56E-20
DENND4B	0.42415059	7.34E-23	7.04E-20
KIF4A	0.42339564	8.89E-23	8.15E-20
CIT	0.4224137	1.14E-22	1.00E-19
TRIP13	0.4222103	1.20E-22	1.01E-19
TICRR	0.42068634	1.77E-22	1.43E-19
C3ORF67	0.41867657	2.93E-22	2.11E-19
RAD54L	0.41461519	8.05E-22	5.41E-19
TTLL6	0.41403907	9.28E-22	6.03E-19
CDC45	0.41313864	1.16E-21	7.25E-19
CCNA2	0.41260245	1.32E-21	7.84E-19
POLQ	0.41070539	2.10E-21	1.21E-18
FANCA	0.41028893	2.33E-21	1.26E-18
CENPA	0.41025042	2.35E-21	1.26E-18
KRTAP5-AS1	0.41019826	2.38E-21	1.26E-18
NUF2	0.40903593	3.16E-21	1.63E-18
KIF18B	0.40790303	4.15E-21	2.09E-18
NEK2	0.40712051	5.02E-21	2.45E-18
DLGAP5	0.40704478	5.11E-21	2.45E-18
TACC3	0.40668256	5.57E-21	2.61E-18
ORAI2	0.40491282	8.52E-21	3.82E-18
CDR2L	0.40476147	8.84E-21	3.87E-18
MELK	0.4041679	1.02E-20	4.32E-18
ESPL1	0.40412705	1.03E-20	4.32E-18
TRIM65	0.4040058	1.06E-20	4.35E-18
TLCD3B	0.40352886	1.19E-20	4.78E-18
FOXM1	0.40139048	1.97E-20	7.78E-18
KIF20A	0.40028375	2.56E-20	9.73E-18

**Table 5:** Top 15 clusters with the representative enriched terms by Metascape (one per cluster).

Description	Log(P-value)	Log(q-value)	Symbols
mitotic sister chromatid segregation	-18.279253	-13.960	CDC20,CENPE,NEK2,PLK1,TRIP13,ESPL1,DLGAP5,KIF14,TACC3,SPAG5,KIF4A,NUF2,KIF18B,FANCA,MYBL2,RAD54L,CENPA,KIF20A,GTSE1,TTLL6,CCNA2,CIT
mitotic cell cycle phase transition	-13.475754	-10.165	CCNA2,CDC20,CENPE,FOXM1,NEK2,PLK1,CDC45,TRIP13,ESPL1,DLGAP5,MELK,KIF14,TACC3,GTSE1,TICRR,KIF20A,SPAG5,CIT,FANCA,RAD54L,NUF2
Cell Cycle, Mitotic	-11.173079	-8.089	CCNA2,CDC20,CENPA,CENPE,FOXM1,MYBL2,NEK2,PLK1,CDC45,ESPL1,KIF20A,GTSE1,NUF2,DLGAP5,KIF14,SPAG5,CIT
mitotic cytokinesis	-8.5636069	-5.789	CENPA,PLK1,ESPL1,KIF20A,CIT,KIF4A,KIF14,KIF18B,TTLL6
PID FOXM1 PATHWAY	-8.1071909	-5.344	CCNA2,CENPA,FOXM1,NEK2,PLK1,MELK,KIF14,GTSE1,TICRR,MYBL2,CDC20,CDC45,ESPL1
microtubule-based movement	-6.2211541	-3.618	CENPE,DLGAP5,KIF14,KIF20A,KIF4A,KIF18B,TTLL6,ORAI2,DENND4B,CDC20
positive regulation of cell cycle process	-6.0735162	-3.495	CDC45,ESPL1,DLGAP5,KIF14,SPAG5,CIT,GTSE1,CCNA2
PID AURORA A PATHWAY	-4.6729241	-2.308	CENPA,DLGAP5,TACC3
regulation of microtubule-based process	-4.3749415	-2.056	NEK2,PLK1,TACC3,SPAG5,TTLL6
DNA repair	-4.3251004	-2.019	FANCA,FOXM1,CDC45,RAD54L,TRIP13,POLQ,TICRR,TCF3,CCNA2,NEK2
positive regulation of mitotic cell cycle phase transition	-3.2047892	-1.003	CDC45,ESPL1,DLGAP5,NEK2
Mitotic G1 phase and G1/S transition	-2.6548733	-0.491	CCNA2,MYBL2,CDC45
regulation of Ras protein signal transduction	-2.4031838	-0.248	FOXM1,DENND4B,KIF14,CCNA2
neuron apoptotic process	-2.0631828	0.000	MYBL2,KIF14,CIT
HTLV-I infection	-2.0017617	0.000	CDC20,MYBL2,TCF3

## Abbreviations

CELSR3: Cadherin EGF LAG Seven-Pass G-Type Receptor 3
PCa: prostate cancer
PCTA: Prostate Cancer Transcriptome Atlas
GS: gleason score
MCODE: molecular complex detection
PSA: prostate specific antigen
OS: overall survival
DFS: disease-free survival
