# Supplementary material for: Increased expression of CELSR3 indicates a poor prognostic factor for Prostate Cancer
Source: J Cancer. 2021 Jan 1;12(4):1115–24. doi: 10.7150/jca.49567 (PMC7797646; doi:10.7150/jca.49567)
Supplement: Supplementary file 1 — Supplementary table. [file jcav12p1115s1.pdf]

Table S1 the sequence of primers.

Gene	Primer sequence
CELSR3	
Forward	CCCACCCCAAAGATGTGGAT
Reverse	GGCTGGTTGTTGTTAGCTGC
GAPDH	
Forward	TCGGAGTCAACGGATTTGGT
Reverse	TTCCCGTTCTCAGCCTTGAC
CENPE	
Forward	ACTGCTCTCCAGTTTGCCAG
Reverse	GGTCACCAGCATCCGTGTTA
CENPA	
Forward	TATTGGCCCTACAAGAGGCAG
Reverse	GCCAGTTGCACATCCTTTGG
CDC20	
Forward	TCGCATCTGGAATGTGTGCT
Reverse	CGGGATGTGTGACCTTTGAGT
NUF2	
Forward	ACTGTAGGTGAGCGCGAGA
Reverse	GCAAGACTTCAGGCTTTGGA
ESPL1	
Forward	ACTGGTCAGGCGGTTAAGTC
Reverse	CTGGAGGGTTGGACAGGAAC
PLK1	
Forward	CCGCAATTACATGAGCGAGC
Reverse	AGGAGACTCAGGCGGTATGT
